# The power and limitations of gene expression pathway analyses toward predicting population response to environmental stressors

**DOI:** 10.1111/eva.12935

**Published:** 2020-03-03

**Authors:** Brenna C.M. Stanford, Danielle J. Clake, Matthew R.J. Morris, Sean M. Rogers

**Affiliations:** ^1^ Department of Biological Sciences University of Calgary Calgary AB Canada; ^2^ Department of Biology Ambrose University Calgary AB Canada; ^3^ Bamfield Marine Sciences Centre Bamfield BC Canada

**Keywords:** adaptation, gene expression, gene networks, pathway analysis, phenotypic plasticity, population persistence, stickleback, temperature tolerance

## Abstract

Rapid environmental changes impact the global distribution and abundance of species, highlighting the urgency to understand and predict how populations will respond. The analysis of differentially expressed genes has elucidated areas of the genome involved in adaptive divergence to past and present environmental change. Such studies however have been hampered by large numbers of differentially expressed genes and limited knowledge of how these genes work in conjunction with each other. Recent methods (broadly termed “pathway analyses”) have emerged that aim to group genes that behave in a coordinated fashion to a factor of interest. These methods aid in functional annotation and uncovering biological pathways, thereby collapsing complex datasets into more manageable units, providing more nuanced understandings of both the organism‐level effects of modified gene expression, and the targets of adaptive divergence. Here, we reanalyze a dataset that investigated temperature‐induced changes in gene expression in marine‐adapted and freshwater‐adapted threespine stickleback (*Gasterosteus aculeatus*), using Weighted Gene Co‐expression Network Analysis (WGCNA) with PANTHER Gene Ontology (GO)‐Slim overrepresentation and Kyoto Encyclopedia of Genes and Genomes (KEGG) pathway analysis. Six modules exhibited a conserved response and six a divergent response between marine and freshwater stickleback when acclimated to 7°C or 22°C. One divergent module showed freshwater‐specific response to temperature, and the remaining divergent modules showed differences in height of reaction norms. *PPARAa,* a transcription factor that regulates fatty acid metabolism and has been implicated in adaptive divergence, was located in a module that had higher expression at 7°C and in freshwater stickleback. This updated methodology revealed patterns that were not found in the original publication. Although such methods hold promise toward predicting population response to environmental stressors, many limitations remain, particularly with regard to module expression representation, database resources, and cross‐database integration.

## 1 INTRODUCTION

Rapid environmental changes predicted by climate models will impact the global distribution and abundance of species, highlighting the urgency to understand and predict how populations will adapt or perish under changing environments (Somero, 2010). The mechanisms by which species respond to, tolerate, and mitigate these changes are fundamentally important toward understanding ecological and evolutionary processes (Bernatchez et al., 2010; Dennenmoser, Vamosi, Nolte, & Rogers, 2017; Yeaman et al., 2016). With the advent of sequencing technologies, there is unprecedented ability to elucidate the molecular variation that explains the visible phenotypes on which selection and evolution ultimately act (Dalziel, Rogers, & Schulte, 2009; Todd, Black, & Gemmell, 2016). While genome sequencing has allowed questions based around population structure, adaptation, and speciation to be resolved at the level of DNA (e.g., Dennenmoser et al., 2017; Rogers & Bernatchez, 2007), it is ultimately how the genome is expressed over time and space that invokes cellular to organism‐wide changes (Pavey, Bernatchez, Aubin‐Horth, & Landry, 2012; Pfennig & Ehrenreich, 2014) upon which evolution can act. Gene expression is shaped by both genetic and environmental components and is considered a molecular phenotype (Aubin‐Horth & Renn, 2009; Pavey, Nosil, & Rogers, 2010). Incorporating molecular phenotypes therefore provides an opportunity to test hypotheses on how evolution has shaped the expression of genes to result in adaptation and persistence to novel environments (Gibbons, Metzger, Healy, & Schulte, 2017; Hoke, Adkins‐Regan, Bass, McCune, & Wolfner, 2019; Pfennig & Ehrenreich, 2014; Schneider & Meyer, 2017).

Changes in gene regulation have long been hypothesized to play a crucial role in driving rapid evolutionary changes (Britten & Davidson, 1969; Saetre et al., 2004; Schulte, 2001; St‐Cyr, Derome, & Bernatchez, 2008). To investigate gene expression as altered by some factor(s) of interest, quantification of RNA content is widely used as a proxy, though it actually captures the dynamic outcome of RNA production, longevity, and degradation**.** Differential expression (DE; the up‐ or down‐regulation of a gene in respect to another environment, phenotype, etc.) is frequently used to determine genes that may be correlated to and possibly be adaptive in response to the condition of interest. DE has been used to investigate questions in a broad array of systems and evolutionary applications (e.g., Christie, Marine, Fox, French, & Blouin, 2016; Harrison, Hammond, & Mallon, 2015). However, while DE analysis results in a list of genes that may be associated with a given phenotype or factor, the results typically provide limited clear inference into biological functions or pathways. It is also becoming increasingly evident that gene products (mRNA and functional noncoding RNA (Gerstein et al., 2007)) often work in conjunction with each other (de la Fuente, 2010; Khatri, Sirota, & Butte, 2012) in a carefully regulated manner that contributes to the tight fits between organisms and their environment—and these noncoding RNA may not be measured by gene expression methodologies. Additionally, DE depends on the hypothesis that the most evolutionarily important genes will be the most expressed, which has been shown not to be the case, especially for key regulators (Hudson, Dalrymple, & Reverter, 2012). Finally, because DE analysis does not necessarily capture all the genes underlying the response to the factor, it makes it increasingly difficult to connect the genes into biologically meaningful explanations (Hudson et al., 2012).

Pathway analyses may help connect the relationship between molecular phenotypes, biological processes, environmental changes, and adaptive phenotypes (Gollery et al., 2006; Hudson et al., 2012; Khatri et al., 2012). These methods group genes that respond similarly to a factor of interest, based on the hypothesis that clustered genes may belong to common pathways and be regulated by the same transcriptional network (van Dam, Võsa, Graaf, Franke, & Magalhães, 2017; Eisen, Spellman, Brown, & Botstein, 1998; Ihmels, Levy, & Barkai, 2004; St‐Cyr et al., 2008). Pathway analyses span a broad range of techniques—including functional classification of genes from different databases (e.g., The Gene Ontology (GO) resource containing annotations deduced primarily by sequence similarity to model organisms (Ashburner et al., 2000; Carbon et al., 2019) and PANTHER GO‐Slim database representing curated, evolutionarily conserved GO annotations; Mi, Muruganujan, Ebert, Huang, & Thomas, 2019), construction of gene modules by grouping genes whose expression are positively or negatively correlated to each other across factors of interest (e.g., Weighted Gene Co‐expression Network Analysis (WGCNA); Langfelder & Horvath, 2008), and detection of known pathways which represent the current knowledge of how gene products interact, including repressors and transcription factors (e.g., The Reactome Knowledgebase; Fabregat et al., 2018 and Kyoto Encyclopedia of Genes and Genomes (KEGG) database; Kanehisa, Sato, Kawashima, Furumichi, & Tanabe, 2016; Ogata et al., 1999). Many of these methods (especially GO term assignment) are frequently used in conjunction with each other and with DE analysis. For example, Filteau, Pavey, St‐Cyr, and Bernatchez (2013) used WGCNA and GO terms in lake whitefish (*Coregonus clupeaformis*) to show that bone morphogenetic protein and calcium signaling may be conserved mechanisms that rapidly evolve in response to trophic behavior, while Healy, Bryant, and Schulte (2017) coupled DE with GO terms and KEGG pathway analysis to illustrate that different mitochondrial genotypes may have limited influence in killifish (*Fundulus heteroclitus*) response to cold acclimation. Additionally, WGCNA has been shown to be powerful in capturing coordinated, low‐level changes across hundreds of genes in response to a stressor where DE analysis failed to detect enough genes to establish biological inference (Orsini et al., 2018; Stanford & Rogers, 2018). However, functional annotation of the genes in these modules is still limited by current knowledge (e.g., Orsini et al., 2018; Rose, Seneca, & Palumbi, 2016).

Overall, while such pathway analyses have shown promise, testing hypotheses related to the molecular mechanisms that facilitate ecological divergence and how populations respond to environmental stressors remain relatively limited. As gene expression is impacted by many internal and external factors which makes elucidating the response to the factor of interest difficult, the combination of controlled common garden experiments in association with a ubiquitous environmental stressor under an integrated framework of elucidating pathways may be better suited to reveal the underlying biological inference of population responses. This is particularly important as environmental stressors can invoke genome‐wide changes in gene expression (Orsini et al., 2018; Rose et al., 2016); pathway analyses can reduce the complexity from thousands of genes that are responsive to a factor to a few hundred groups or pathways (Khatri et al., 2012).

Temperature, an “abiotic master factor” (Hall, Stanford, & Hauer, 1992), is an environmental stressor that influences physiology, behavior, and morphology and can induce changes in these associated traits (phenotypic plasticity) (Angilletta, 2009) affecting the distribution and abundance of organisms (Reynolds & Casterlin, 1979; Sunday, Bates, & Dulvy, 2012; Viña, 2002). Many organisms, especially ectotherms, are particularly vulnerable due to their reliance on environmental temperature (Jarrold et al., 2019). Temperature has been observed to simultaneously alter the expression of thousands of genes and different pathways (Healy et al., 2017; Long et al., 2013; Metzger & Schulte, 2018) and has shaped gene expression plasticity in response to temperature, activating or suppressing key genes and pathways (Fuentes, Zuloaga, Valdes, Molina, & Alvarez, 2014). This has resulted in adaptive variation among species and ecotypes residing in different thermal regimes (Todgham, Hoaglund, & Hofmann, 2007; Tschantz, Crockett, Niewiarowski, & Londraville, 2002). Yet, the extent to which populations can adaptively respond to forecasted changes in temperature is largely unknown (Donelson et al., 2019; Somero, 2010; Sunday et al., 2012).

Threespine stickleback (*Gasterosteus aculeatus*) have experienced widespread postglacial colonization of freshwater lakes and streams from marine habitats, with marine populations thought to closely resemble the ancestral state prior to the last ice age (Bell, 1977; Hohenlohe et al., 2010; Walker & Bell, 2000; but see Morris, Bowles, Allen, Jamniczky, & Rogers, 2018) in contrast to derived freshwater populations that exhibit adaptive evolution in response to environmental stressors such as salinity change (Gibbons et al., 2017; McCairns & Bernatchez, 2010), competition/predation (Rogers et al., 2012), and different thermal regimes (Barrett et al., 2011; Gibbons, Rudman, & Schulte, 2016; Morris et al., 2014). Lakes often have warmer summer temperatures and colder winter temperatures compared to marine habitats, which has been reflected in freshwater stickleback evolving larger thermal windows (Smith Wuitchik, 2019; Barrett et al., 2011). However, this change in thermal limit can be rapidly induced, with marine stickleback evolving cold tolerance similar to freshwater stickleback within three generations in semi‐natural pools (Barrett et al., 2011) and within a generation when acclimated to simulated freshwater winter temperature (Gibbons et al., 2016). Plasticity in gene expression also likely plays a role in freshwater sticklebacks' responses to a thermally variable habitat. Derived freshwater stickleback populations have increased plasticity in gene expression compared to marine populations when acclimated to temperatures close to their thermal tolerance (Morris et al., 2014). This provides evidence that plasticity may be necessary to adaptation to novel environment, though it should be noted that suppression of plastic response may also be selected for (e.g., Morris & Rogers, 2013; Velotta, Ivy, Wolf, Scott, & Cheviron, 2018). Additionally, Morris et al. (2014) found that seven of these differentially plastic genes showed overlap with regions of divergence between freshwater and marine stickleback found in Jones et al. (2012) (Morris et al., 2014). Yet, despite the intensive research toward understanding the role of temperature on gene expression and physiological changes, there has been relatively little application linking these to the functional pathways that bridge the two that may facilitate evolutionary resilience and recovery.

The objective of this study was to test the hypothesis that ecotype‐specific responses to temperature, as initially revealed through differential expression analysis, can be related to the modification of key biological processes and the pathways that underlie them. To do this, we first reanalyzed an experimental dataset that tested genomic norms of reaction in response to temperature for two replicate populations of freshwater and marine threespine stickleback (Morris et al. (2014). Given that Morris et al. (2014) found support for the hypothesis that derived stickleback ecotypes evolved increased plasticity in response to temperature, we predicted that relevant gene modules (i.e., groups of potentially interacting genes) should exhibit parallel responses to temperature across replicate populations of the same ecotype. We tested these predictions with three predominant methods: WGCNA (Langfelder & Horvath, 2008) with PANTHER GO‐Slim term (Mi et al., 2019) and KEGG pathway overrepresentation for the modules (Kanehisa et al., 2016; Ogata et al., 1999). We predicted (a) that GO term enrichment would return more cohesive biological processes as they were run on data‐driven modules of co‐responding genes rather than a list of DE genes and that there would be less terms returned when using annotations of higher quality; (b) that KEGG pathways relating to the GO terms would be enriched providing better insight into the mechanisms behind how the genes are connected; and (c) that adaptive divergence at seven genes known to differentiate ecotypes would be associated with those modules and pathways showing different responses between freshwater and marine stickleback. Finally, we discuss these results in the context of promises and limitations of these analyses and what is needed to overcome the challenges they pose in inferring the role and biological inference of differential gene expression in evolutionary applications.

## 2 METHODS

### 2.1 Experimental design

The experimental design and data generation were conducted as per Morris et al. (2014). Briefly, threespine stickleback (*Gasterosteus aculeatus*) were collected from two freshwater (Cranby Lake, Texada Island, 49°42,000″, 124°30,000″ and Hoggan Lake, Gabriola Island, 49°36,000″, 124°01,020″) and two marine (Oyster Lagoon, 49°36,048.6″N, 124°1,046.88″ and Little Campbell River, 49°104″, 122°45,052″) locations on the coast of British Columbia. Pure F1 families of ancestral marine and derived freshwater stickleback were generated and maintained at 17–18°C as eggs and juveniles and were then divided into 7°C or 22°C treatments, representing temperatures close to their thermal limit. All fish were kept at 5–6 ppt. Multiple replicates per treatment were maintained in these conditions for 1,700 growing degree days. Two fish per population from each replicate were sampled (44 total). RNA was extracted from white muscle (a tissue with well‐established plasticity necessary for response to environment; Johnston, 2006) with dermal and skeletal tissue using TRIzol (Invitrogen)‐chloroform (Chomczynski & Sacchi, 1987). cDNA was hybridized to the fully vetted, species‐specific threespine stickleback microarray (Leder, Merilä, & Primmer, 2009), with one array per individual. Each microarray contained replicates of 20,021 unique transcripts totaling 61,662 control and feature spots (details in Morris et al., 2014). Linear Model for Microarrays (LIMMA, Smyth, 2004, 2005) software was used to perform between‐array normalization using the quantile method on log2‐transformed signal intensities. High‐intensity features were kept for further analysis if they passed threshold (>2.6 *SD*) on 85% of the 7 or 22°C arrays. After filtering, 14,208 genes remained that form the basis of this analysis.

### 2.2 Gene module construction

In preparation for using the normalized microarray data for network analysis, missing values were imputed through nearest neighbor averaging using the *impute.knn* function of the impute package in R (v 1.58; Hastie et al., 1999; Troyanskaya et al., 2001). Gene networks were created using the WGCNA package in R (v 1.68; Langfelder & Horvath, 2008), as follows. The *cor* function was used to obtain Pearson's correlation for patterns of co‐expression between genes across samples. Soft thresholding power was chosen using the power estimate from the *pickSoftThreshold* function and the network inferred from co‐expression data. Modules were detected by first building a topological overlap matrix from expression adjacencies (using the *TOMsimilarity* function), converting to a distance matrix, and building hierarchical clusters using the *hclust* function in the fastcluster package (Müllner, 2013). Clusters were dynamically cut using the *cutreeDynamic* function in the dynamicTreeCut package (Langfelder, Zhang, & Horvath, 2008). Finally, close modules were merged using the *mergeCloseModules* function in WGCNA. After modules were created and probes assigned to them, the eigengene (first principal component) of each module was calculated using the *moduleEigengenes* function of WGCNA.

### 2.3 Association of modules to temperature and habitat

Two‐way ANOVAs were used to determine associations between module eigengene expression and experimental factors (rearing temperature (7 or 22°C), habitat (freshwater or marine), and the interaction terms). Two‐way ANOVAs were calculated for each module with both type II and type III sums of squares using the car package in R (Fox and Weisberg 2011). Where there was no significant interaction, the effect of each treatment was determined from Bonferroni‐corrected (Bonferroni 1936) type II sums of squares. We selected a conservative threshold to focus on the most significant results but without necessarily capturing the breadth of genes responding to the treatment. In modules where the interaction term was significant, the effects of individual treatments were tested using Bonferroni‐corrected type III sums of squares. Interaction significance was then assessed for all modules after Bonferroni correction. Modules showing significant differences in eigengenes across temperature, habitat, and/or the interaction after correction were selected for further analysis.

### 2.4 GO term enrichment for significant modules

Genes contained within significant modules were prepared for Gene Ontology (GO) (Ashburner et al., 2000; Carbon et al., 2019) term enrichment by first converting threespine stickleback Ensembl transcript IDs to PANTHER IDs using the *getBM* function in the R biomaRt package (v 2.40.5; Smedley et al., 2015). These PANTHER IDs were then loaded into PANTHER (http://pantherdb.org/ v 14.1; (Mi et al., 2019; Thomas et al., 2003), and a statistical overrepresentation test was run using PANTHER GO‐Slim Biological and PANTHER GO‐Slim Molecular process annotation datasets (released: 2019‐03‐12) (Mi et al., 2019). Each module was tested against a reference list consisting of the PANTHER IDs for the 14,208 genes that passed filtering and tested using Fisher's exact test (Fisher 1936) and corrected for multiple correction using Bonferroni correction.

### 2.5 KEGG pathway enrichment for significant modules

Threespine stickleback Ensembl transcript IDs for the module genes were converted to human and zebrafish (*Danio rerio*) NCBI Entrez IDs for compatibility in Kyoto Encyclopedia of Genes and Genomes (KEGG) (Kanehisa et al., 2016; Ogata et al., 1999) pathway enrichment (KEGG does not recognize ENSEMBL IDs). This conversion was done using the function *getLDS* in the R biomaRt package (v 2.40.5; Smedley et al., 2015). Because there is not a 1:1 mapping of threespine stickleback Ensembl transcript IDs to Entrez Gene IDs (multiple Ensembl IDs map to a single Entrez ID and vice versa), we first created a reference list to convert threespine stickleback Ensembl ID (14,208 genes) to Entrez IDs, removing any ambiguous mapping IDs.

Genes from each significant module were converted to human and zebrafish Entrez IDs using the above reference list. The *enrichKEGG* function in the R package clusterProfiler (v 3.12.0; Yu, Wang, Han, & He, 2012) was used to enrich for KEGG pathways with Bonferroni correction for all significant modules. Three of these KEGG pathway were used in downstream analyses described below.

### 2.6 Mapping expression onto three KEGG pathways

We selected three significantly enriched pathways to visualize the expression profiles for the genes in those pathways (Bonferroni‐corrected *p*‐value < .05). We endeavored to select pathways previously demonstrated to be involved in temperature acclimation of fish (Healy et al., 2017; Long et al., 2013; Metzger & Schulte, 2018; Scott & Johnston, 2012). We selected spliceosome and ribosome biogenesis and oxidative phosphorylation. Log_2_ differential expression was calculated for freshwater and marine stickleback setting 22°C as the baseline (positive values thus indicating up‐regulation of genes at 7°C). Differential expression was visualized using the *pathview* function in the R package Pathview (v 1.24.0; Luo & Brouwer, 2013). For spliceosome and ribosome genes, freshwater and marine stickleback showed similar patterns of differential expression and freshwater stickleback expression profiles were arbitrarily picked over marines to be displayed. For oxidative phosphorylation, freshwater and marine stickleback exhibited different expression changes between 22°C and 7°C and both are displayed.

### 2.7 Assigning genes observed in divergent regions between freshwater and marine stickleback to modules and pathways

Morris et al. (2014) discovered seven genes (NOD‐like receptor family CARD domain containing 5 (*NLRC5*), peroxisome proliferator‐activated receptor alpha a (*PPARAa*), inhibin, alpha (*INHa*), obscurin‐like 1 (*OBSL1*), insulin‐like growth factor binding protein 2a (*IGFBP2a*), *SPEG,* and a novel gene) that showed differential expression in response to temperature and corresponded to outlier regions of adaptive divergence between freshwater and marine stickleback found by Jones et al. (2012). To investigate the association of these regions to module and pathway analysis, we determined which module these genes were assigned to and whether they were found in significantly enriched human and/or zebrafish KEGG pathways.

## 3 RESULTS

### 3.1 Sixteen modules were associated with temperature, habitat, and/or the interaction

Of the 20,021 genes represented on the microarray, 14,208 passed normalization and filtering. These genes were used to construct the modules with WGCNA. Thirty‐four modules were detected (named, in order of most to least genes, ME01 to ME34), ranging in size from 20 genes to 4,752. Of these modules, 16 (total of 10,335 genes; Table 1) were significantly associated with temperature, habitat, and/or the interaction between them after Bonferroni correction (*p* < .05) (Table 1).

**Table 1:** Number of genes and response of modules significantly associated with temperature, habitat, and/or the interaction between temperature and habitat

Module	Number of genes	Temperature	Habitat	Temperature*Habitat	Response
ME01	4,752	x			Higher at 7°C vs. 22°C
ME02	1,791	x	x		Freshwater and marine both lower at 7°C. Freshwater higher than marine at both 7°C and 22°C
ME04	693	x		x	Freshwater and marine similar at 22°C, both higher at 7°C but freshwater more so
ME05	565	x			Higher at 7°C vs. 22°C
ME06	471		x		Freshwater higher than marine across temperatures
ME09	389	x	x		Higher at 7°C. Freshwater higher than marine at both temperatures
ME10	364	x	x		Higher at 7°C. Freshwater higher than marine at both temperatures
ME11	334	x			Higher at 7°C vs. 22°C
ME12	289		x		Freshwater lower than marine across temperatures
ME14	204	x			Higher at 7°C vs. 22°C
ME17	164		x		Freshwater lower than marine across temperatures
ME22	99	x	x		Higher at 7°C vs. 22°C. Freshwater lower than marine across temperatures
ME24	82		x		Freshwater lower than marine across temperatures
ME26	66	x			Lower at 7°C vs. 22°C
ME29	48	x	x		Higher at 7°C vs. 22°C. Freshwater lower than marine across temperatures
ME33	24	x			Lower at 7°C vs. 22°C

### 3.2 Six modules show response to temperature independent of habitat

Modules ME01, ME05, ME11, ME14, ME26, and ME33 were found to have eigengenes that differed between temperature treatments similarly for both freshwater and marine stickleback (Table 1). At 7°C, four modules (ME01, ME05, ME11, and ME14) had higher eigengenes and two modules (ME26 and ME33) had lower eigengenes when compared to 22°C (Table 1).

### 3.3 Five modules responded to temperature and habitat

Modules ME02, ME09, ME10, ME22, and ME29 had eigengenes that differed between temperature treatments and also exhibited different levels of expression between freshwater and marine stickleback (Table 1). One module (ME02) had a lower eigengene at 7°C compared to 22°C, with freshwater stickleback having higher values across both temperatures (Table 1). Four modules had higher eigengenes at 7°C compared to 22°C, where freshwater stickleback had higher eigengenes at both temperatures for two modules (ME09 and ME10) and lower eigengenes across both temperatures for two modules (ME22 and ME29) compared to marine stickleback (Table 1).

### 3.4 One module shows response to temperature in a habitat‐dependent manner

Module ME04 was found to have eigengenes that differed between temperature treatments in a habitat‐dependent manner (Table 1). Freshwater and marine eigengenes were similar at 22°C with the eigengene for freshwater stickleback increasing at 7°C (Table 1).

### 3.5 Four modules responded to habitat in a temperature‐independent manner

Module ME06 had higher eigengenes across temperatures in freshwater stickleback compared to marine stickleback (Table 1). Modules ME12, ME17, and ME24 had lower eigengenes across temperatures in freshwater stickleback compared to marine stickleback (Table 1).

### 3.6 GO term enrichment success was variable across modules

Over 94% of genes (9,871 of 10,335) were successfully linked to a GO term using PANTHER IDs; however, ~60% did not belong to a GO term that has of yet been deemed to be evolutionarily conserved (“unclassified”) in the PANTHER GO‐Slim databases (Table S1). Though each module contained genes with GO‐Slim term assignment, significant overrepresentation after Bonferroni correction of GO terms varied across modules, with 43.75% (seven modules: ME02, ME05, ME09, ME12, ME17, ME29, and ME33) exhibiting no significant enrichment of molecular or biological processes (Table 2). The remaining modules had overrepresentation for either molecular processes (ME06, ME11,and ME24), biological processes (ME01 and ME22), or both (ME04, ME10, ME14, and ME26) (Table 2). Using PANTHER GO‐Slim biological processes to detect evolutionarily conserved GO terms in modules associated with temperature returned 35 overrepresented biological processes (Fishers' exact test with Bonferroni correction *p* < .05; Table 2). Morris et al. (2014) found there to be 48 and 54 enriched GO terms for freshwater and marine stickleback, respectively, for genes up‐regulated at 7°C and 22 and 10 for genes up‐regulated at 22°C (Fishers' exact test *p* < .001).

**Table 2:** GO term enrichment and KEGG pathway enrichment for the modules significantly associated with temperature, habitat, and/or the interaction between temperature and habitat

Module	Biological process GO‐slim terms	Molecular process GO‐slim terms	Human KEGG pathways	Zebrafish KEGG pathways
ME01	Organic substance metabolic process, ribonucleoprotein complex biogenesis, metabolic process	No terms enriched	Spliceosome, ribosome biogenesis in eukaryotes, RNA transport, fatty acid metabolism, valine, leucine and isoleucine degradation, proteasome, DNA replication, RNA polymerase, mismatch repair, peroxisome, amino sugar and nucleotide sugar metabolism, carbon metabolism, terpenoid backbone biosynthesis	Ribosome biogenesis in eukaryotes, spliceosome, RNA transport, DNA replication, mismatch repair, RNA polymerase, terpenoid backbone biosynthesis
ME02	No terms enriched	No terms enriched	Proteoglycans in cancer	No pathways enriched
ME04	Skeletal system development	Extracellular matrix structural constituent	RNA degradation, protein digestion and absorption, ribosome	RNA degradation, ribosome
ME05	No terms enriched	No terms enriched	Human T‐cell leukemia virus 1 infection, longevity regulating pathway, Fc gamma R‐mediated phagocytosis	No pathways enriched
ME06	No terms enriched	Structural constituent of ribosome	Ribosome	Ribosome, mitophagy, autophagy
ME09	No terms enriched	No terms enriched	No pathways enriched	No pathways enriched
ME10	Oxidation–reduction process, cellular respiration, energy derivation by oxidation of organic compounds, ATP synthesis coupled proton transport, energy coupled proton transport down electrochemical gradient, proton transmembrane transport, oxidative phosphorylation, ATP metabolic process, inorganic cation transmembrane transport, ion transmembrane transport, inorganic ion transmembrane transport, aerobic respiration, ribose phosphate metabolic process, purine ribonucleotide metabolic process, ribonucleotide metabolic process, mitochondrial ATP synthesis coupled electron transport, ATP synthesis coupled electron transport, metabolic process	Structural constituent of ribosome Cofactor binding, proton transmembrane transporter activity, proton‐transporting ATP synthase activity rotational mechanism, electron transfer activity	Oxidative phosphorylation, thermogenesis, Parkinson disease, Huntington disease, nonalcoholic fatty liver disease, Alzheimer disease, ribosome, retrograde endocannabinoid signaling, citrate cycle, fatty acid degradation, carbon metabolism	Oxidative phosphorylation, ribosome
ME11	No terms enriched	Phosphatase regulator activity	MAPK signaling pathway	mRNA surveillance pathway, ubiquitin‐mediated proteolysis
ME12	No terms enriched	No terms enriched	No pathways enriched	No pathways enriched
ME14	DNA metabolic process, cellular macromolecule biosynthetic process, DNA biosynthetic process, DNA‐dependent DNA replication, DNA replication, nucleic acid metabolic process, cellular biosynthetic process, cellular response to stress	DNA binding, catalytic activity, acting on DNA, heterocyclic compound binding, sequence‐specific double‐stranded DNA binding	DNA replication, mismatch repair, nucleotide excision repair	DNA replication, nucleotide excision repair, mismatch repair, pyrimidine metabolism
ME17	No terms enriched	No terms enriched	No pathways enriched	No pathways enriched
ME22	Intracellular signal transduction, signal transduction, cellular response to stimulus	No terms enriched	Hepatocellular carcinoma	No pathways enriched
ME24	No terms enriched	Oxidoreductase activity, acting on paired donors, with incorporation or reduction of molecular oxygen	No pathways enriched	No pathways enriched
ME26	Response to cAMP, response to organic cyclic compound, response to mechanical stimulus	Proximal promoter sequence‐specific DNA binding, RNA polymerase II proximal promoter sequence‐specific DNA binding	Osteoclast differentiation	No pathways enriched
ME29	No terms enriched	No terms enriched	No pathways enriched	No pathways enriched
ME33	No terms enriched	No terms enriched	No pathways enriched	No pathways enriched

The amount of overrepresented processes varied among modules. For example, modules ME01 and ME10 were significantly enriched for three and 18 biological processes (Fishers' exact test with Bonferroni‐corrected *p*‐value < .05), respectively (Table 2). Broadly related molecular and biological processes were observed within a module (e.g., ATP synthesis in ME10, DNA‐related processes in ME14).

### 3.7 Human and zebrafish show similar KEGG pathway enrichment

There were 9,683 genes (68.2%) and 10,861 (76.44%) of the 14,208 stickleback ENSEMBL IDs that unambiguously converted to human Entrez ID and zebrafish Entrez IDs, respectively (Table S1). For the significant modules, 7,257 of 10,335 genes (72.8%) mapped to a human Entrez ID and 8,035 of 10,335 genes (77.7%) mapped to a zebrafish Entrez ID (Table S1).

Less than half of the genes in significant modules that unambiguously mapped to an Entrez ID have been mapped into a KEGG database pathway (42.69% (3,098 of 7,257genes) for human Entrez IDs and 34.45% (2,770 of 8,035 genes) for zebrafish Entrez IDs) (Table S1). Six modules (ME09, ME12, ME17, ME24, ME29, and ME33) did not have enrichment for human pathways and ten modules (ME02, ME05, ME09, ME12, ME17, ME22, ME24, ME26, ME29, and ME33) did not reveal significant enrichment for zebrafish pathways (Table 2). There was similarity in the pathways enriched between humans and zebrafish. For example, in ME01, all the pathways found with zebrafish Entrez IDs are also found using human Entrez IDs, with an additional five pathways (Table 2). This module in both cases seems to be enriched for pathways relating to transcription and translation. For ME10, using the human Entrez IDs resulted in human disease state pathways. Perhaps the most different pathways between the mapped ID types are seen in ME11, where human Entrez IDs returned MAPK signaling and zebrafish Entrez IDs returned mRNA surveillance and ubiquitin‐mediated proteolysis (Table 2).

### 3.8 Majority of genes up‐regulated on two pathways in me01

**Figure 1:**
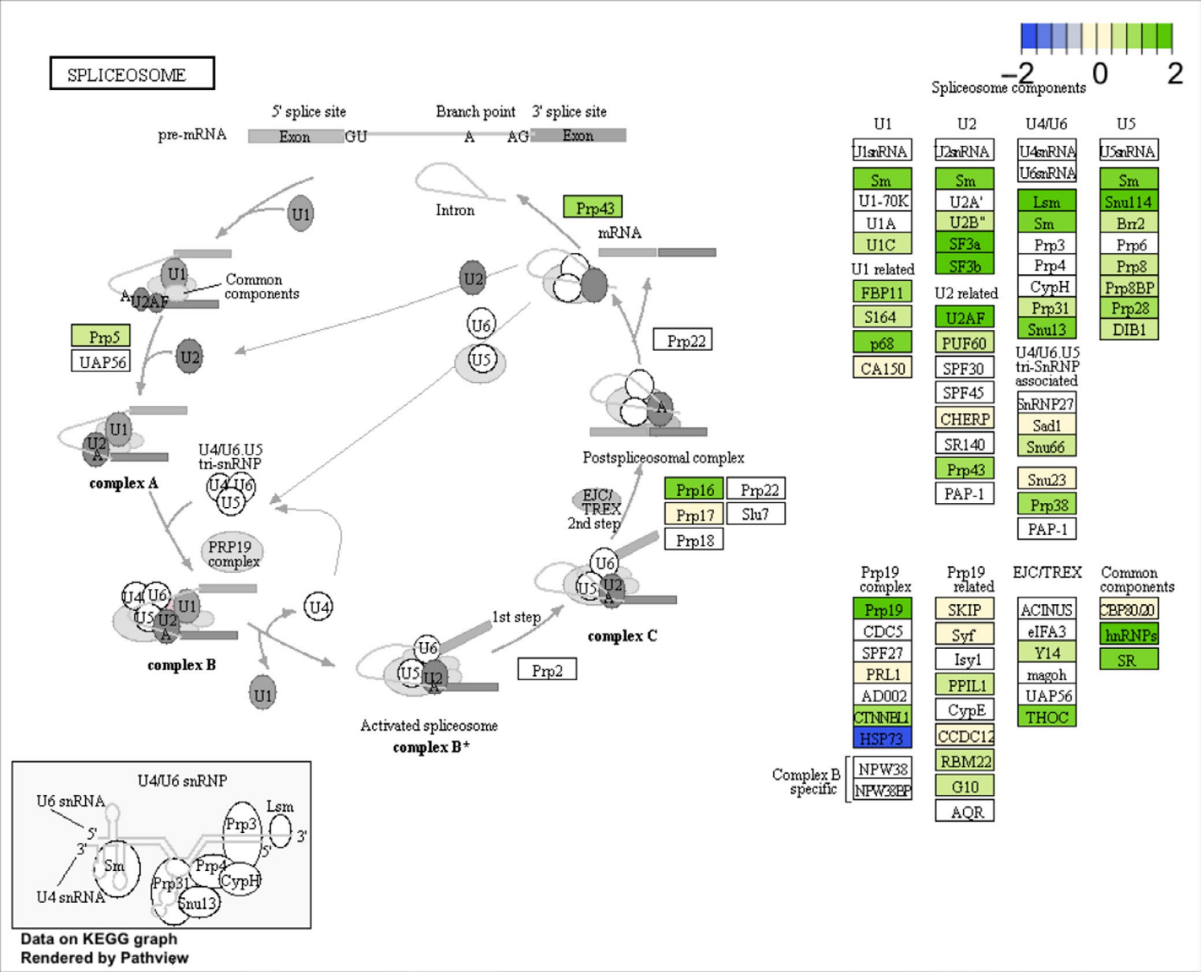
The spliceosome pathway (hsa/dre03040) as enriched in module ME01 (Bonferroni correction < .05). Freshwater and marine stickleback exhibited similar expression differences between 7°C and 22°C. Shown is freshwater stickleback log_2_ differential expression where green indicates an up‐regulation at 7°C, blue indicates a down‐regulation at 7°C, cream indicates no change in expression between 7°C and 22°C and white means that no information for that gene was present

The two most enriched pathways in module ME01 (spliceosome: hsa/dre03040 and ribosome biogenesis: hsa/dre03008) showed patterns of up‐regulation at 7°C in both freshwater and marine individuals. For the spliceosome, expression at 7°C for the mapping genes shows that 40 genes were up‐regulated, five genes showed no change, and three genes were down‐regulated when compared to 22°C (Figure 1). For the ribosome biogenesis pathway at 7°C, 30 genes were up‐regulated, six genes showed no change, and three genes were down‐regulated when compared to 22°C (Figure S1).

### 3.9 Freshwater and marine stickleback exhibit different responses to temperature in expression of genes involved in the oxidative phosphorylation pathway

For the genes mapping to the oxidative phosphorylation pathway (hsa/dre00190), all but one gene (cytochrome c oxidase assembly factor heme A:farnesyltransferase; *COX10* or *cyoE*) showed up‐regulation at 7°C for freshwater stickleback (Figure S2). In marine stickleback, almost half of the genes showed no change in expression (including *COX10*), one showed slight down‐regulation (NADH dehydrogenase (ubiquinone) 1 beta subcomplex subunit 9 (*NDUFB9*)), and the remaining showed some up‐regulation (Figure S3).

### 3.10 Module association of plasticity genes implicated in adaptive divergence

Six of the seven genes with divergent plasticity found by Morris et al. (2014) were found in modules associated with temperature, habitat, and/or the interaction. Three of the genes were found in modules associated with temperature and habitat (*NLRC5* and *INHa* in ME09 and *PPARAa* in ME10) (Table 3). *IGFBP2a* was included in ME04, which is associated with temperature and the interaction between temperature and habitat (Table 3). The novel gene and *OBSL1a* were found in habitat associated modules, ME06 and ME12, respectively (Table 3). The remaining gene, *SPEGa,* was found in a module without significant association (ME16) (Table 3). *PPARAa* was the only gene found in an enriched KEGG pathway (hsa04932: nonalcoholic fatty liver disease) (Table 3).

**Table 3:** Module and KEGG pathway membership of the six genes found to have plastic expression located in regions of divergence

Gene name	Gene abbr.	Habitat	Response of DE in found in Morris et al. (2014)	Module associated	Human Entrez ID	Zebrafish Entrez ID	Human KEGG pathways	Zebrafish KEGG pathways
NOD‐like receptor family CARD domain containing 5	*NLRC5*	Marine	Down‐regulated at 7°C	9	84166	Not found	Not found	Not found
Peroxisome proliferator‐activated receptor alpha a	*PPARAa*	Freshwater	Up‐regulated at 7°C	10	5465	563298	Nonalcoholic fatty liver disease (hsa 04932)	Not found
Inhibin, alpha	*INHa*	Freshwater	Down‐regulated at 7°C	9	3623	570520	Not found	Not found
Obscurin‐like 1	*OBSL1*	Freshwater	Down‐regulated at 7°C	12	Not found	796577	Not found	Not found
Insulin‐like growth factor binding protein 2a	*IGFBP2a*	Freshwater	Down‐regulated at 7°C	4	3485	794176	Not found	Not found
SPEG	*SPEG*	Freshwater	Down‐regulated at 7°C	16	Not found	570504	Module not significantly associated with temperature or habitat	
Novel gene	*Novel gene*	Freshwater	Down‐regulated at 7°C	6		Not applicable	Not applicable	Not applicable

## 4 DISCUSSION

The objective of this study was to test the hypothesis that, if temperature has induced evolutionarily adaptive change at the level of the molecular phenotype, this will be reflected in the modification of key biological processes and the pathways that underlie them. We found that modules of co‐expressed genes detected by WGCNA revealed conserved and divergent responses to temperature between marine and freshwater stickleback. Enriching these modules for GO terms reduced the number of biological GO terms compared to DE analysis alone, organizing many into groups of functionally related processes. KEGG pathway enrichment further refined the results, organizing related genes into the same pathway. These analyses provide novel insights to the potential mechanisms that underlie the evolutionary changes that differentiate marine and freshwater stickleback ecotypes in response to temperature.

### 4.1 Almost 10,000 genes are correlated with temperature treatment

Temperature induced responses in eigengene expression in 12 modules that included 9,329 of the 14,208 genes analyzed (66%). These results are consistent with temperature as a master factor, whereby one of the key characteristics of molecular responses to temperature change is that it routinely causes a response from a large portion of the genome across taxa and tissue types (Gierz, Forêt, & Leggat, 2017; Rosell et al., 2014; Seneca & Palumbi, 2015; Yakovlev et al., 2014). Indeed, across fish species, shifts in expression have been observed in dramatic numbers of genes (Healy et al., 2017; Long et al., 2013; Metzger & Schulte, 2018; Morris et al., 2014). This is perhaps not surprising when the range of biological processes that are impacted by temperature in an ectotherm are considered—from enzymatic catalytic function (Kohen & Klinman, 1998), protein structure integrity (Somero, 1995), metabolic rate (Clarke & Johnston, 1999), and cell membrane lipid re‐modeling (Hazel, McKinley, & Gerrits, 1998), to organism‐wide changes such as growth (Jonassen, Imsland, & Stefansson, 1999), swimming ability (Wang, Tan, Jiao, You, & Zhang, 2014), and thermoregulatory behavior (e.g., Smith Wuitchik 2019). WGCNA has the power to identify modules of genes that exhibit coordinated patterns of expression, where the module is then tested for significance to treatments and is therefore not limited to genes detected as differentially expressed (Orsini et al., 2018). This may explain the increased number of genes observed to be associated with temperature in this study (over 60%) compared to the ~20 to 40% of the transcriptome exhibiting differential expression in response to temperature reported by Healy and Schulte (2019), although their inclusion of alternative splicing and use of RNA‐sequencing data (vs. microarray) may be influencing this comparison. This is a distinct advantage in the detection of regulatory genes (e.g., transcription factors) which show lower levels of expression than nonregulatory genes (Vaquerizas, Kummerfeld, Teichmann, & Luscombe, 2009).

The approach of grouping genes into modules was more informative for biological inference and pathways compared to the original study of Morris et al. (2014). Of all the 16 modules, module ME01 contained the greatest number of genes (almost 50% of the genes sorted into modules) and was observed to be induced at 7°C in a conserved manner between marine and freshwater stickleback. This module was enriched primarily for biological processes and pathways involved in RNA manufacturing, transport and modification, and protein translation. Since modules can include both down‐ and up‐regulated genes (though an increased eigengene does indicate overall higher levels of expression) (Langfelder & Horvath, 2008), we investigated the expression profiles for the two most enriched pathways (spliceosome and ribosome biogenesis) in ME01 more closely. These pathways were observed to have similar patterns of expression between marine and freshwater stickleback, which was a general up‐regulation at 7°C. Up‐regulation of genes at colder temperatures involved in these processes is observed in many species of fish (e.g., larval zebrafish (Long et al., 2013), killifish (Healy et al., 2017), threespine stickleback (Metzger & Schulte, 2018) but see Fuentes et al., 2014 in carp (*Cyprinus carpio*). It is also frequently seen that cold acclimation induces more genes than warm acclimation (e.g., Healy et al., 2017; Morris et al., 2014; Scott & Johnston, 2012; Healy & Schulte, 2019; but see Metzger & Schulte, 2018) and rate of protein synthesis has been demonstrated to decrease in cold‐acclimated spotted wolffish (*Anarhichas minor* Olafsen) (Lamarre, Le Frangois, Driedzic, & Blier, 2009). Cold‐adapted Antarctic fish have higher levels of ubiquitin‐conjugated proteins (marking them for degradation) (Todgham et al., 2007), and longjaw mudsuckers (*Gillichthys mirabilis*) exhibit increased levels of protein degradation when cold‐acclimated (Somero & Doyle, 1973). Potentially, up‐regulation of mRNA transcripts and protein synthesis machinery may be necessary to ensure appropriate amounts of functional proteins. Cold acclimation has also been shown to result in alternative splicing of mRNA, which may explain the up‐regulation of the spliceosome pathway (Healy & Schulte, 2019). Morris et al. (2014) found enrichment of GO terms related to RNA processing and tRNA metabolic processes in genes up‐regulated at 7°C in marine and freshwater stickleback and RNA modification and translational initiation in marine and freshwater, respectively. While these GO terms are related to the functions shown in module ME01, use of WGCNA coupled with KEGG pathway analysis was observed to create functional groupings of pathways that aided in detection of biological signal. However, ultimately, more research is necessary to explain how the patterns of gene expression observed here and across fish species potentially facilitate species persistence to cold.

### 4.2 Freshwater stickleback may have increased plasticity in gene expression

Freshwater and marine stickleback exhibited different eigengene expression in six modules that were responsive to temperature. One module (ME04), that contained almost 700 genes, was determined to have a significant interaction between temperature and habitat representing a freshwater stickleback‐specific response to cold acclimation (limitations of detection for the other modules discussed below). This module was enriched for the GO biological process term skeletal system development and KEGG pathways involved in protein and RNA degradation and the subunits of the ribosome, effectively compressing these genes into a few processes and pathways (Khatri et al., 2012). None of the genes predicted to be under selection between freshwater and marine stickleback (from past genome‐wide outlier analysis by Jones et al., 2012 and exhibiting plastic expression in Morris et al., 2014) were observed in this module.

Although freshwater stickleback may have evolved greater plasticity, as represented by module ME04, it also seems that the evolution of the height of reaction norms (Castric, Billiard, & Vekemans, 2014) has been an important component of freshwater stickleback evolution to temperature. Four of the six modules showed higher eigengene values at both temperatures for freshwater compared to marine stickleback, and two modules (ME22 and ME29), enriched for cell signaling and response processes, appear to be down‐regulated in freshwater stickleback. Although several of these modules were not enriched for biological processes and/or pathways, module ME10 appears to be involved in modification of energy production. It would seem reasonable then that although both ecotypes respond to temperature by up‐regulating genes at 7°C, freshwater stickleback have evolved the capacity to boost transcription above marine levels at cold temperatures, but only by shifting their reaction norms rather than changing the slope. It is unclear how this shift would correspond to fitness at 22°C, as both ecotypes presumably have similar critical thermal maxima (Barrett et al., 2011). Importantly, only WGCNA analysis with GO enrichment could elucidate this evolutionary process.

### 4.3 Limitations of module analysis

Eigengene values endeavor to capture the expression profile of hundreds or thousands of genes in a single value while explaining as much variation as possible. As such, they may not accurately represent individual gene's or pathway's expression profile changes (Langfelder & Horvath, 2008). For instance, module ME10 was significantly associated with temperature and habitat but not the interaction implying there was no habitat‐specific response to temperature. However, further investigation of the oxidative phosphorylation pathway showed that freshwater and marine stickleback exhibited different levels of fold change in gene expression response at 7°C, illustrating how the eigengene lacks resolution, and more detailed investigation of the pathways contained is necessary. The oxidative phosphorylation pathway is comprised of five complexes which ultimately lead to the synthesis of ATP from products generated from glycolysis, fatty acid oxidation, and the citric acid cycle (Chaban, Boekema, & Dudkina, 2014). As catalytic rate of enzymes is decreased at colder temperature, a potential strategy to cope seen in cold‐adapted fish is to up‐regulate mRNA and protein translation for key enzymes (O'Brien, 2011; Somero, 1995). Freshwater stickleback have increased cytochrome c oxidase (COX; 1.9.3.1) activity (characterized by the amount of substrate turned over per minute per gram of wet tissue) in response to cold acclimation, likely due to an increase in the amount of COX present in the cell (Orczewska, Hartleben, & O'Brien, 2010). Enzyme activity has be shown to be variable between related species and between enzymes within a species in response to acclimation temperature indicating that even within closely related species, thermal response may evolve independently (Tschantz et al., 2002). Altogether, this illustrates that while eigengene values are appropriate for associating modules to treatments, further analysis may be necessary to detect shifts in expression in individual processes and pathways.

### 4.4 The limitations of GO term analyses

Many of the molecular and biological processes arising from GO analyses assume an independence between genes (Khatri et al., 2012) that is unlikely to exist. For instance, though module ME10 was observed to have 18 biological processes associated with it, they arise due to multiple related GO terms which are nested under each other in the GO Tree, such as metabolic process > ATP metabolic process > oxidative phosphorylation > ATP synthesis coupled electron transport (http://www.informatics.jax.org/vocab/gene_ontology/GO:0008152).

There remain limitations when trying to determine and interpret the annotation for protein coding genes responding to temperature. GO term annotations in nonmodel organisms are frequently deduced from regions of sequence similarity with annotated genes in model species (Pavey et al., 2012), and function is not confirmed by experiments (Gaudet, Livstone, Lewis, & Thomas, 2011). Here, we used a carefully curated database that only includes GO term functions that are judged to be evolutionarily conserved (Mi et al., 2019). While only ~40% of the GO terms assigned to the genes of interest had inferred function, the database is continually expanding to annotate and organize more genes (Mi et al., 2019). However, the functions vary in specificity of molecular or biological process (e.g., 28 genes in ME01 mapped to the same molecular function GO term for GTPase activity (GO:0,003,924)). One would not necessarily expect to have an “enrichment” for GO terms that share domains such as these, as the genes are not necessarily in the same pathway or even activated in response to the same stimuli. For instance, GTPase is a large superfamily of proteins (Bourne, Sanders, & McCormick, 1991), which play integral roles in the communication of signals from the cell membrane and belong to various pathways and regulate specific biological processes (Bourne et al., 1991; Neves, Ram, & Iyengar, 2002). This is not an inherent problem to GO terms but rather that detailed annotation of genes, especially in nonmodel organisms, is limited. Many taxonomically conserved genes of interest (e.g., crustacean specific stress response in Orsini et al., 2018) lack sequence similarity to a characterized domain, which prevents assignment of even predicted molecular function (Bossi et al., 2017; Gollery et al., 2006).

### 4.5 Limitations of kegg pathway analysis

In contrast to GO terms, KEGG is a carefully curated database that represents the best in our current knowledge of gene pathways (Kanehisa et al., 2016; Ogata et al., 1999). Less than half of the genes in the significant modules reported above have been included in pathways, which does not imply that the remaining genes are not important, but rather that they represent the limits in our current understanding. For example, peroxisome proliferator‐activated receptor alpha a (*PPARAa*) is a gene that has been implicated in freshwater and marine stickleback divergence (Morris et al., 2014) and is also induced by cold in zebrafish (Scott & Johnston, 2012). However, though zebrafish have been proposed as a model to research *PPARAa's* role on adipogenesis and obesity (Den Broeder, Kopylova, Kamminga, & Legler, 2015), it has yet to be fit into any pathways for zebrafish on KEGG. However, it has been fit into seven human pathways, including the enriched pathway nonalcoholic fatty liver disease, though KEGG does not indicate how genes are involved in disease states (Khatri et al., 2012). *PPARAa* (orthologous with *PPARA* in humans; Den Broeder et al., 2015) is a transcription factor expressed in cells with high levels of fatty acid oxidation, including skeletal muscle tissue (Loviscach et al., 2000; Pawlak, Lefebvre, & Staels, 2015), that regulates the expression of enzymes involved in this process, though fatty acid metabolism was sorted into module ME01. Transgenic mice over‐expressing *PPARA* were observed to have increased fatty acid oxidation rates, decreased glucose uptake, and, interestingly, up‐regulation of mRNA for components of the oxidative phosphorylation pathway (Finck et al., 2005). *PPARA* is related to nonalcoholic fatty liver disease because deficiency in *PPARA* leads to lipid accumulation in the liver and researchers have proposed up‐regulation of its activity may be an effective treatment for nonalcoholic fatty liver disease (Pawlak et al., 2015). While *PPARA* has been well characterized in mouse and human models, there remains limited information on the pathways it is involved in on KEGG, decreasing the usefulness of this database in uncovering its role in adaptive divergence between freshwater and marine stickleback.

KEGG assumes independence between pathways and will return pathways that share an overlapping gene set (Khatri et al., 2012), which was observed for several of the pathways in module ME10. The assumption of independence requires the researcher to be aware that enrichment of certain pathways and GO terms does not necessarily mean a stronger signal for related processes.

We also encountered lack of integration across gene databases, which resulted in the exclusion of thousands of potentially interesting genes from further analysis. With increasing number of expression studies and increased prevalence of noncoding RNA which are often classed as “novel transcripts” though they have been characterized in a different database, methods that address convergence of databases are necessary (Weirick, John, & Uchida, 2017).

### 4.6 Moving forward

Our knowledge of how evolution shapes molecular phenotypes to respond to environmental change has grown considerably in recent years. In this study, we illustrated how pathway analyses can increase biological inference in regard to potentially adaptive differences between freshwater and marine stickleback in response to temperature. To continue linking these to adaptive differences, we must now focus on understanding the functional molecular mechanisms underlying such adaptive evolution (Dalziel et al., 2009). Newer methods are investigating the clusters and networks themselves, with the general idea of searching biological pathways for subnetworks of genes that directly interact with each other and that present unusual evolutionary features (Gouy, Daub, & Excoffier, 2017; Rougeux, Gagnaire, Praebel, Seehausen, & Bernatchez, 2019). Interrupting candidate genes (such as *PPARAa*) via CRISPR (Wucherpfennig, Miller, & Kingsley, 2019) or modifying their expression via small, interfering RNA (Giacomotto, Rinkwitz, & Becker, 2015) could help infer molecular function, providing better annotation (a limitation detected in this study) and aid their integration into pathways. For example, transgenic stickleback have been used to show that regulatory changes in pituitary homeobox 1 (*PITX1*) underlie the repeated evolution of pelvic reduction (Chan et al., 2010), that changing levels of ectodysplasin (*EDA*) directly impact plate development (Colosimo et al.., 2005), and that parallel evolution in DNA regulation allows freshwater stickleback to alter *EDA* expression in armor plates (O'Brown, Summers, Jones, Brady, & Kingsley, 2015). Increased knowledge about the importance of regulatory noncoding RNA in the evolution of more complex traits (Barrett, Fletcher, & Wilton, 2012; Mattick, 2004; Taft, Pheasant, & Mattick, 2007) has seen the creation of innovative databases (e.g., the ZFLNC (Hu et al., 2018) and RegenDbase (King et al., 2018)), which will be helpful to fill in gaps in our current regulatory pathway knowledge. Pairing sequencing methods, such as ATAC‐seq which returns the level of accessibility across the genome (Buenrostro, Wu, Chang, & Greenleaf, 2015), could present a link to understanding plasticity in the ecology and evolution of gene expression. While we frequently focus on gene expression (as in this manuscript), mRNA abundance is a proxy for protein abundance, influenced by post‐transcriptional and post‐translational level regulation (Payne, 2015; Vogel & Marcotte, 2012). Proteomics is necessary to determine whether the alteration in RNA content (as seen in the three pathways explored here) results in increased proteins (Payne, 2015; Vogel & Marcotte, 2012) and integrating metabolomics would help determine the biochemical outcomes of these changes (Lankadurai, Nagato, & Simpson, 2013). Ultimately, we must endeavor to link molecular phenotypes to observable phenotypes, such as thermal performance curves (Schulte, 2015), measures of fitness (e.g., reproductive fitness (Schreck, 2010)), and swimming ability (Scott & Johnston, 2012) to elucidate how evolution has acted on these molecular mechanisms to facilitate population persistence and evolution. As Louis Bernatchez has championed, an integrated approach to studying these questions will help in achieving these goals (Box 1).

BOX 1

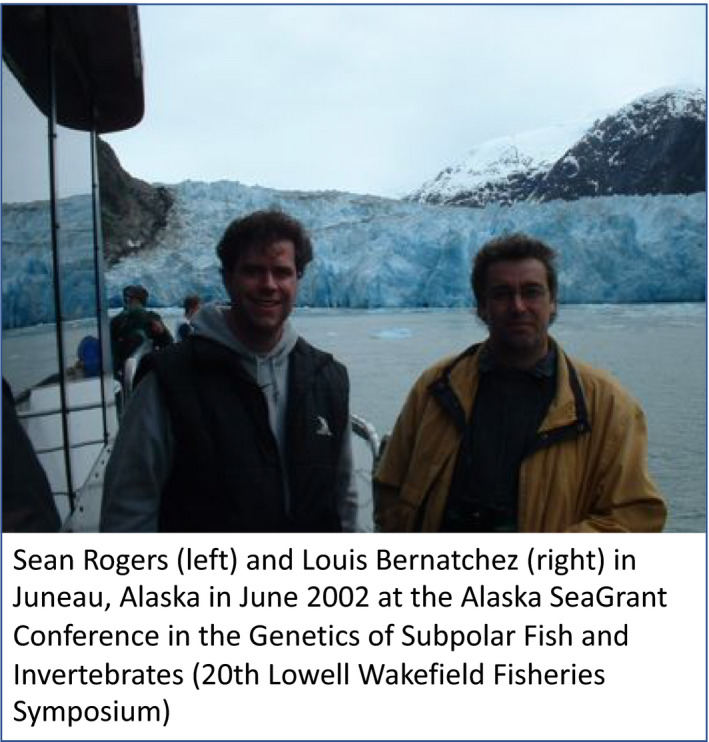

My first memory of Louis stems from an email I sent to him in 1996 (using PINE) asking for assistance in scoring the “SFO‐23 microsatellite locus”. True to form, he replied right away, telling me “I do not have time for your interrogations”. I was sure that my aspirations of working with Louis were over, but relieved to eventually learn that “interrogations” does not translate directly to English very well.

Louis has always had a large, dynamic group that was an exhilarating research environment for his students. The combination of field work, wet lab facilities in the world renowned LAboratoire de Recherche en Sciences Aquatiques (LARSA), and molecular ecology lab (at a time when the field of Molecular Ecology was just taking off) at Université Laval was more than a new PhD student could dream for.

Personally, I have always been amazed at Louis' capacity to advance research in ecology and evolution in a myriad of important ways. Part of the inspiration for our present contribution stemmed from a conversation I remember having with Louis in 2005 when he was preparing for one of his invited seminar presentations. Louis placed a lot of importance on providing new data and ideas in his talks, and encouraged this practice with his students. In this particular presentation, Louis' background information included King & Wilson's (1975) influential study arguing that protein comparisons between human and chimpanzee showed too few differences to account for their observed phenotypic differences, speculating that this diversity may be explained by the evolution of gene regulation. Of course, Louis went on to examine how quantitative variation in gene expression relates to phenotypic variation in a variety of adaptive traits, with several influential contributions from his group in the lake whitefish system that have since found tremendous support for this hypothesis, while highlighting that we still have much to learn with respect to the evolution of gene regulation. In this regard, Louis has always encouraged his students to be avid readers of the literature, keep an open mind and observe when it comes to biological variation in all forms, and be fearless when it comes to new areas of inquiry – especially when you are doing something you enjoy that improves science and society. The co‐authors in this contribution reveal that his philosophy will continue to inspire future generations of scientists and conservation practitioners.

## CONFLICT OF INTEREST

None Declared.

## Data Availability

The data that support the findings of this study are available from Morris et al. (2014) in Dryad at https://doi.org/10.5061/dryad.5q65k.
