# Supplementary figures and images for: The power and limitations of gene expression pathway analyses toward predicting population response to environmental stressors
Source: Evol Appl. 2020 Mar 3;13(6):1166–82. doi: 10.1111/eva.12935 (PMC7359838; doi:10.1111/eva.12935)

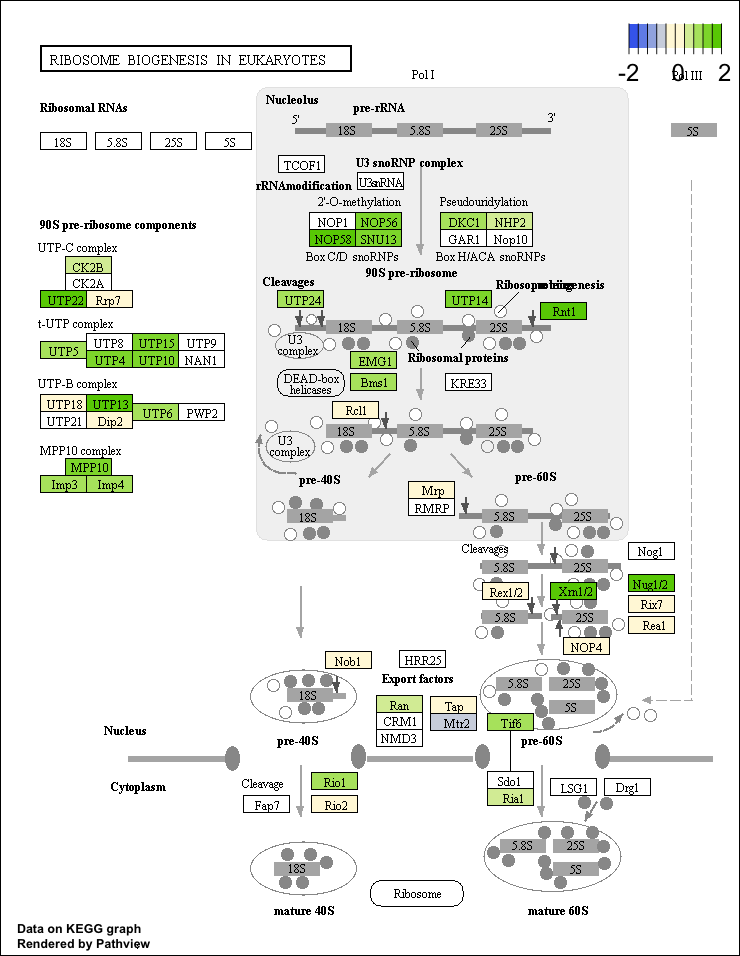

Supplement: Supplementary file 1 [file EVA-13-1166-s001.png]

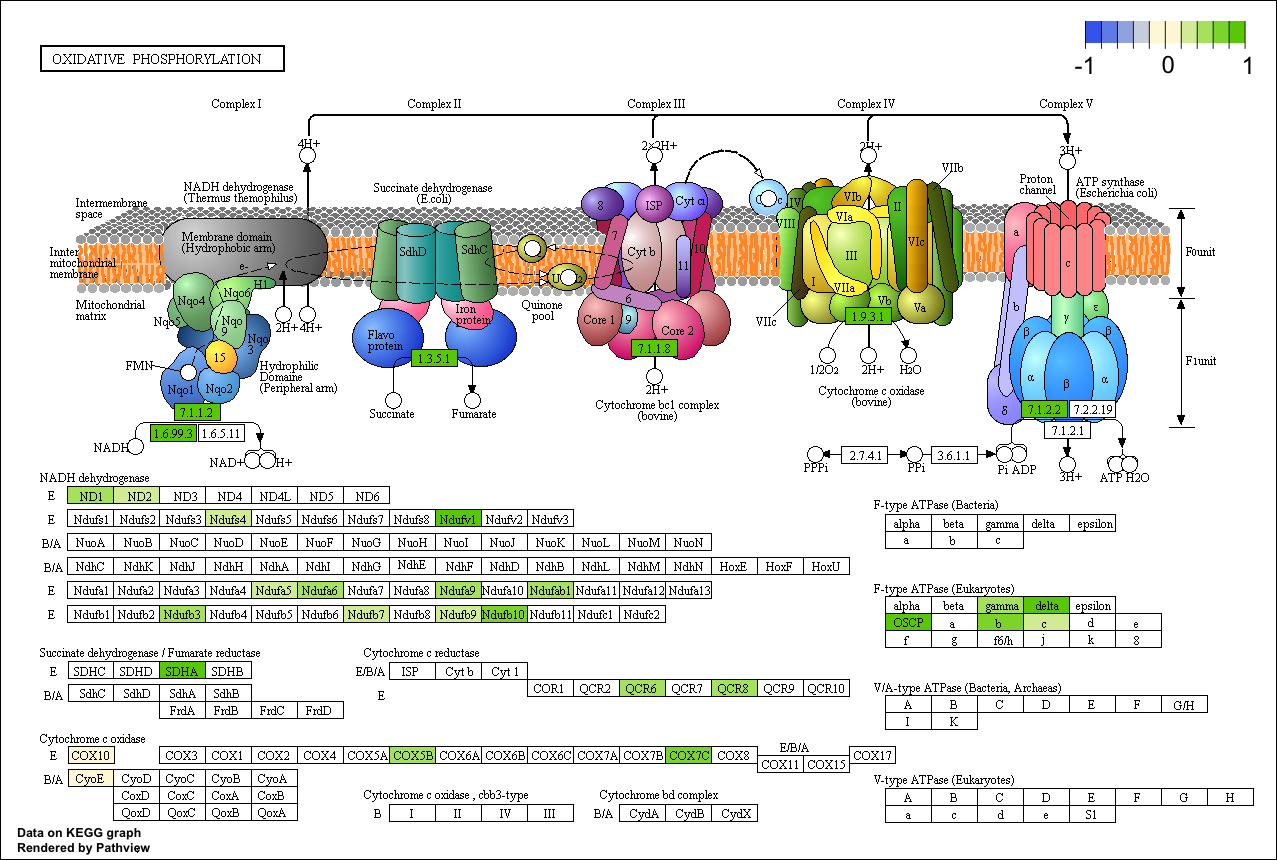

Supplement: Supplementary file 2 [file EVA-13-1166-s002.png]

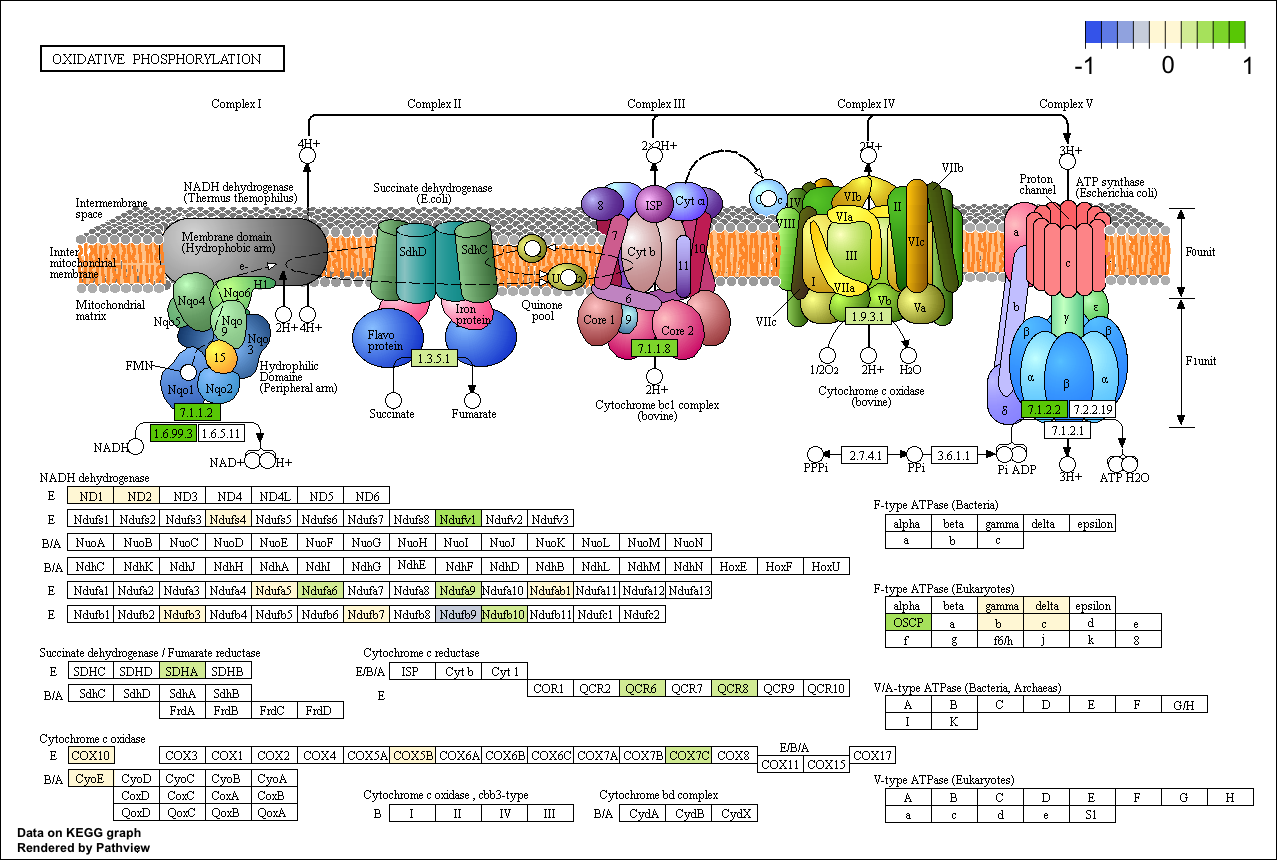

Supplement: Supplementary file 3 [file EVA-13-1166-s003.png]
